# How do advanced diagnostics support public health policy development?

**DOI:** 10.2807/1560-7917.ES.2019.24.4.1900068

**Published:** 2019-01-24

**Authors:** Jacob Moran-Gilad

**Affiliations:** 1 Dept. of Health Systems Management, School of Public Health, Faculty of Health Sciences, Ben Gurion University of the Negev, Beer Sheva, Israel; 2 ESCMID Study Group for Genomic and Molecular Diagnostics, Basel, Switzerland

**Keywords:** advanced diagnostics, whole genome sequencing, MALDI-TOF MS

Microbiologists working in clinical/diagnostic microbiology or public health microbiology (mainly food, water and environmental), have experienced a major revolution of their profession over recent years. Technological advancements involving the development and implementation of new analytical platforms have allowed for faster, more accurate and more complex diagnostics [1]. Some of these technologies are novel and emerge as ‘disruptive technologies’, while others improve and enhance existing diagnostic approaches. In this context, how do we define ‘advanced diagnostics’?

Advanced diagnostics can be divided into several groups, according to their methodological approach as well as their practical applications. One such division differentiates between culture-dependent (culture-based) and culture-independent microbiology (Table). With culture-based diagnostics, applicable mainly to bacterial and fungal pathogens, one or more culture phases are involved in order to yield growth of the suspected microorganism from a clinical or non-clinical sample. Subsequently, growing isolates are characterised with respect to taxonomy, antimicrobial drug susceptibility and other traits (such as virulence and molecular subtypes) by a range of approaches. These mainly include—but are not necessarily restricted to—characterisation by conventional (phenotypic) techniques, molecular assays targeting specific genes, proteomics (primarily taxonomical identification using matrix-assisted laser desorption-ionisation time-of-flight mass spectrometry (MALDI-TOF-MS)) or single-cell whole genome sequencing (WGS), followed by bioinformatics analyses to call the taxonomy and phylogenomic subtype and infer phenotypic resistance and virulence, by mapping the resistome and virulome. WGS, powered by next-generation sequencing (NGS), is undoubtedly the most impactful application, downstream to culture isolation, and has the potential to serve as a one-stop-shop for pathogen characterisation, while allowing for unprecedented accuracy and resolution [2].

**Table:** Advanced diagnostics by technology and approaches, 2019

Approach	Technology							
	Conventional / standard microbiology	Molecular microbiology		Proteomics	Molecular standard typing methods	Genomics / metagenomics		
		PCR	Multiplex PCR	MALDI-TOF-MS		WGS	Microbiomics	Whole genome metagenomics
Culture-based	Organism ID/AST	Detection/Sanger sequencing of specific gene for characterisation of grown organism (e.g. resistance or virulence determinant)	Detection of specific genes for characterisation of grown organism (e.g. resistance or virulence determinant),	Identification of grown organism; more recently, potential for detection of resistance or typing	PFGE, SLST, MLST, MLVA	ID/AST, mapping of resistome and virulome, typing by SNPs or cgMLST	NA	NA
Culture- independent	NA	Detection of specific genes, for organism presence (or characteristic such as presence of specific gene)	Syndromic testing for a range of potential pathogens per sample type	Application of MALDI-TOF-MS directly on samples still experimental	NA	NA	Microbial population analysis	Microbial population analysis, functional characterisation, extraction of whole genome assemblies, phenotype prediction

AST: antimicrobial susceptibility testing; cgMLST: core genome multilocus sequence typing; ID: identification; MALDI-TOF MS: matrix-assisted laser desorption ionization-time of flight mass spectrometry: MLST: multilocus sequence typing; MLVA: multilocus variable number tandem repeat analysis; NA: not applicable; PFGE: pulsed-field gel electrophoresis; SLST: singlelocus sequence typing; SNP: single nucleotide polymorphism; WGS: whole genome sequencing.

On the other hand, culture-independent microbiology involves the application of diagnostic techniques directly on clinical or non-clinical samples, while obviating the need to recover an organism by culture. This approach has long been used in the field of virology, where virus isolation is rarely performed for routine diagnostic purposes whereas it was not common practice for other pathogens. However, culture-independent detection methods are also applicable to bacterial, fungal and parasitic diseases. With culture-independent microbiology, several diagnostic strategies are now commonly used also for the latter group of pathogens, including the application of PCR assays targeting specific genes that relate to presence of a pathogen and/or an important inferred phenotype, such as antimicrobial resistance to a key agent. More recently, a massive increase in the availability of in-house and commercial multiplex PCR assays is evident, covering a wide range of diagnostic targets in a single run. These assays are increasingly designed for syndromic diagnosis, covering the most common pathogens causing infection in well-defined infectious disease syndromes such as respiratory, gastrointestinal or genitourinary syndromes, as well as syndromes caused by central nervous system infections and even bloodstream infections [3]. Rapid diagnostic tests (RDTs) that are derivatives of syndromic multiplex assays have been designed to generate rapid results in a fairly robust manner and they could be used outside the medical laboratory, closer to the patient or in the field, even by non-laboratorians [4]. These point of care (POC) or point of impact (POI) molecular tests are highly promising also with respect to their impact on public health. Lastly, applying NGS technology directly on samples, an approach also known as metagenomics, has been used for many years now in ecology and environmental sciences. It has the potential, when applied on clinical materials, to accurately map the microbial population in a body site (i.e. the microbiome) by amplification of a target gene such as the 16S rRNA gene, or to generate information regarding the entire taxonomical composition of a sample, while allowing deeper analysis of microbial characteristics and functions (shotgun or whole genome metagenomics) [5]. The latter is especially appealing because of its potential for not only analysing the microbiota, but also allowing whole genome assemblies’ extraction from the metagenome, enabling therapeutic inferences and, in the future, complementary analysis of the host human genome or transcriptome for tailoring treatment and establishing prognosis.

In this special issue of *Eurosurveillance*, 10 articles describe the development and application of such advanced diagnostics, with respect to communicable diseases of public health concern. Through this suite of articles, it is evident that the diagnostic revolution in the field of microbiology is already creating a major impact on public health response and policy making related to infectious diseases.

Two papers focus on harnessing WGS for performing national surveillance of pathogens of public health importance. The first, by Toleman et al., demonstrates the added value of genomic surveillance of meticillin-resistant *Staphylococcus aureus* (MRSA) in the United Kingdom (UK) [6]. This one-year study of all available isolates implicated in bloodstream infections demonstrated the dynamics of MRSA diversity in the UK, identified high-risk clones and contextualised several reported outbreaks. The second paper, by Jenkins et al., shares the UK experience of standardising genomic surveillance of Shiga-toxin producing *Escherichia coli* (STEC) as a foodborne pathogen [7]. This effort proved successful with respect to resolving case clusters with obscure epidemiological data and provided insight into the evolution of pathogenic strain and geographical spread.

Four papers focus on employing WGS for cluster/outbreak investigation in different settings. Fazio et al. studied the increase in serogroup W *Neisseria meningitidis* in Italy over nearly two decades, showing an unusual cocirculation of two meningococcal lineages originating from South America and the Hajj pilgrimage [8]. Similarly, Siira et al. investigated an increase in *Salmonella* Chester infections in Norway also over nearly two decades. WGS dissected this cluster of cases into several distinct geographical origins and unravelled the occurrence of an outbreak originating in another European country [9]. Abascal et al. used WGS to target cross-border surveillance of tuberculosis in Spain. Their data confirm the limitations of the mycobacterial interspersed repetitive-unit-variable-number tandem-repeat (MIRU-VNTR) approach, in that MIRU-VNTR failed to discriminate importations and recent transmissions [10]. Finally, Wüthrich et al. studied an exceedance of legionellosis cases in the city of Basel, Switzerland. Genomic analysis revealed several interesting features, including the contamination of cooling towers by multiple strains, the involvement of highly conserved strains in causing disease over a long time period and the interrelations between cooling towers, which could form a complex microbial network in the same area [11].

Rodriguez-Sánchez et al. reviewed the utility of MALDI-TOF-MS for public health purposes, beyond the main application of proteomics. Such applications include direct application of MALDI-TOF MS on positive blood cultures to improve time to detection of pathogens causing bacteraemia (especially Gram-negative rods), using MALDI-TOF-MS for identification of molecular mechanisms of resistance such as carbapenemases and using MALDI-TOF MS for phylogenetic typing for strains tracking and outbreak detection [12].

Three papers demonstrate the strength of culture-independent microbiology. Ricci et al. performed an evaluation of a commercial and an in-house qPCR assay for the detection of *Legionella pneumophila* in respiratory samples [13]. Their results show that qPCR outperformed the urinary antigen test and culture. While these findings are not unexpected, mindful of the known limitation of these two methods, the increase in sensitivity by molecular diagnosis has public health implications, as more Legionnaires’ disease cases and clusters will be detected and investigated. In another paper, van der Veer et al. report on a culture-independent method they developed for typing *Neisseria gonorrhoeae* [14]. This approach is advantageous, as typing of this fastidious organism requires its isolation in culture, which may be challenging. The method developed and implemented by the authors improved the typeability by ca 50%. Interestingly, this approach has also shown that multiple subtypes may coinfect individuals, which is an important epidemiological finding that would have otherwise been missed, should culture be performed as per existing guidelines from a single anatomical site. Lastly, Kafetzopoulou et al. have used metagenomics to recover the near-full sequences of arboviruses from clinical samples that tested positive for chikungunya or dengue viruses using real-time reverse transcription-PCR [15]. The authors have successfully used two different sequencing technologies. While the samples sequenced were serum/plasma, which are normally sterile, making the bioinformatics analysis for genome recovery less challenging, these findings are encouraging with respect to the feasibility of future metagenomics approaches for arboviral diseases.

Despite the promising results, several challenges remain and need to be addressed by the public health, microbiological and infectious disease communities. Reliance on culture-based methods prolongs the turnaround time for diagnosis and, despite WGS being increasingly streamlined, producing clinically actionable information in real-time via WGS is still challenging. Moreover, predicting phenotypes based on genomics (e.g. prediction of minimum inhibitory concentration to antimicrobials) is still not readily achievable [16]. MALDI-TOF MS has become very popular and many frontline laboratories are using it routinely. Still, more advanced applications of MALDI-TOF MS, such as assessment of antimicrobial resistance or typing, require more development and validation [16]. With culture-independent approaches, multiplex testing may detect non-culturable, non-viable organisms whose significance is unknown, as is the frequent detection of co-infections that are difficult to translate into management decisions while validation is ongoing. Increased reliance on multiplex PCRs also suggests the reduced availability of cultured organisms, which has consequences with respect to strain referral and reference microbiology as a central element of microbiological surveillance at national and international levels. With metagenomics there are still many hindrances, including costs, disparities in capabilities and capacities for performing deep sequencing, optimisation of sample preparation and, most importantly, the bioinformatics analysis, which is incredibly complex, especially when genotype to phenotype correlations are sought.

As proteomics, genomics and metagenomics are increasingly being implemented in microbiology laboratories there are many aspects that need further consideration. These encompass quality control, including the use of certified reference materials and internal and external quality assurance [1,17,18]. Furthermore, there is a need for validation of bioinformatics pipelines that will allow a standardised analysis [19] and meet accreditation requirements, for ensured reverse compatibility between methods [18], for data safety and security, for data sharing agreements as well as deposition and metadata collection etc. The successful implementation of advanced diagnostics in the service of public health, thus depends on many factors. Appropriate national and international frameworks are needed that support timely diagnosis of infectious diseases and high pathogen resolution by using the most appropriate diagnostic methods available today or becoming available in the near future.
